# Bulleyaconitine A Inhibits Morphine-Induced Withdrawal Symptoms, Conditioned Place Preference, and Locomotor Sensitization Via Microglial Dynorphin A Expression

**DOI:** 10.3389/fphar.2021.620926

**Published:** 2021-02-26

**Authors:** Meng-Jing Zhao, Mi-Ya Wang, Le Ma, Khalil Ali Ahmad, Yong-Xiang Wang

**Affiliations:** King’s Lab, Shanghai Jiao Tong University School of Pharmacy, Shanghai, China

**Keywords:** bulleyaconitine A, dynorphin A, microglia, nucleus accumbens, hippocampus, physical dependence, conditioned place preference, locomotor sensitization 3

## Abstract

Bulleyaconitine A (BAA), a C19-diterpenoid alkaloid, has been prescribed as a nonnarcotic analgesic to treat chronic pain over four decades in China. The present study investigated its inhibition in morphine-induced withdrawal symptoms, conditioned place preference (CPP) and locomotor sensitization, and then explored the underlying mechanisms of actions. Multiple daily injections of morphine but not BAA up to 300 μg/kg/day into mice evoked naloxone-induced withdrawal symptoms (i.e., shakes, jumps, genital licks, fecal excretion and body weight loss), CPP expression, and locomotor sensitization. Single subcutaneous BAA injection (30–300 μg/kg) dose-dependently and completely attenuated morphine-induced withdrawal symptoms, with ED_50_ values of 74.4 and 105.8 μg/kg in shakes and body weight loss, respectively. Subcutaneous BAA (300 μg/kg) also totally alleviated morphine-induced CPP acquisition and expression and locomotor sensitization. Furthermore, subcutaneous BAA injection also specifically stimulated dynorphin A expression in microglia but not astrocytes or neurons in nucleus accumbens (NAc) and hippocampal, measured for gene and protein expression and double immunofluorescence staining. In addition, subcutaneous BAA-inhibited morphine-induced withdrawal symptoms and CPP expression were totally blocked by the microglial metabolic inhibitor minocycline, dynorphin A antiserum, or specific KOR antagonist GNTI, given intracerebroventricularly. These results, for the first time, illustrate that BAA attenuates morphine-induced withdrawal symptoms, CPP expression, and locomotor sensitization by stimulation of microglial dynorphin A expression in the brain, suggesting that BAA may be a potential candidate for treatment of opioids-induced physical dependence and addiction.

## Introduction

Opioid addiction is the accumulated results of tolerance and dependence, mainly including physical and psychological dependence (Wang et al., 2019). Physical dependence seeks drug repeatedly and increases gradually doses of the drug to avoid any withdrawal symptoms (Nestler et al., 1993). Psychological dependence refers to drug craving and euphoria achieved by repeated medication, which is difficult to eliminate. Drug relapse can persist for a long time after drug cessation in human being (Robbins et al., 2008) and the high rate of relapse after detoxification is a major clinical problem and becomes a severe challenge to treat drug abuse (Aguilar et al., 2009). Morphine and related opioids are the most potent and widely used analgesics in treating moderate to severe pain. However, development of tolerance and dependence are the important limitation to use of opioid drugs in chronic pain management (Thorn et al., 2016; Ruzza et al., 2019). Opioid addiction in Western countries, particularly in the United States, has become a serious health and social problem in recent years, which requires to be urgently addressed (Wingo et al., 2016; Ezard et al., 2018).

Dynorphin A is widely distributed in the central nervous system to bind to three opioid receptor subtypes with different affinities, especially to κ-opioid receptors (KORs) (Fallon and Leslie, 1986; Schwarzer, 2009). KORs are also widely distributed in the brain (Yuferov et al., 2004; Shippenberg et al., 2007; Bruchas et al., 2010), and the dynorphin/KOR system plays an important role in pain/analgesia, temperature, emotions, and neuroendocrine functions (Pfeiffer et al., 1986; Bodnar, 2010). The dynorphin/KOR pathway is also a major anti-reward system and participates in development of drug addiction. There is growing evidence showing that administration of dynorphin A and other related opioid peptides alleviates withdrawal symptoms of morphine physical dependence (Takemori et al., 1993; Hooke et al., 1995). The KOR agonists, unlike the μ-opioid receptor agonists, do not produce any reinforcing effects but reduce drug abuse under certain conditions. Indeed, it was reported that the KOR agonists ethylketocyclazocine and U50,488 attenuated cocaine behavioral sensitization, conditioned place preference (CPP) acquisition, and self-administration in rhesus monkeys by repressing the release of dopamine (Maisonneuve et al., 1994). It was also reported that the dynorphin/KOR system antagonized the rewarding effects in drug abuse and inhibited the brain reward function by suppressing dopamine release from the mesolimbic reward pathway (Chartoff et al., 2008; Mysels and Sullivan, 2009). On the contrary, it was also reported that the KOR antagonists nor-BNI and arodyn blocked stress-induced reinstatement of cocaine-induced self-administration or CPP acquisition (Beardsley et al., 2005; Carey et al., 2007). These data suggest a complex role of the dynorphin/KOR system in the drug abuse development.

Bulleyaconitine A (BAA), isolated from the rhizomes of *Aconitum bulleyanum*, is a C19-diterpenoid alkaloid without activities of binding to opioid receptors (Wang et al., 2007). As it is a nonnarcotic analgesic and has lower toxicity and wider treatment window than aconitine, BAA has been widely prescribed in China to treat various forms of chronic pain over four decades (Bello-Ramirez and Nava-Ocampo, 2004; Xie et al., 2018). Accumulated evidence demonstrated that BAA and its analogs aconitine (C19-diterpenoid), bullatine A (C20-diterpenoid), and lappaconitine (C18-diterpenoid) produced antinociception without inducing antinociceptive tolerance in various rodent models of pain hypersensitivity, including neuropathic pain, bone cancer pain, inflammatory pain, diabetic pain, and visceral pain (Li et al., 2016a; Li et al., 2016b; Huang et al., 2016; Sun et al., 2018; Huang et al., 2020a; Huang et al., 2020b). Our recent studies further uncovered that BAA, aconitines, bullatine A, and lappaconitine alleviated pain directly through stimulating spinal microglial dynorphin A expression and subsequently activating KORs (Huang et al., 2016; Li et al., 2016a; Li et al., 2017; Sun et al., 2018). In addition, BAA and bullatine A injection blocked chronic morphine-induced antinociceptive tolerance in rats and mice (Li et al., 2016a; Huang et al., 2017a). These studies led to our hypothesis that aconitines including BAA may have a therapeutic potential in treatment of morphine withdrawal symptoms and compulsive drug-seeking and abuse.

In this study, we assessed the inhibitory effects of BAA on regulation of morphine-induced withdrawal symptoms, CPP acquisition and expression, and locomotor sensitization. We first tested whether a subcutaneous BAA injection attenuated naloxone-induced withdrawal symptom in chronic morphine-treated mice. We then assessed whether subcutaneous BAA inhibited morphine-induced CPP acquisition and expression and locomotor sensitization. Thereafter, we explored the involvement of microglial dynorphin A expression and subsequent KOR activation in BAA-induced anti-addictive effects. Our results uncover that BAA inhibits morphine-induced withdrawal symptoms, CPP acquisition and expression, and locomotor sensitization through microglial expression of dynorphin A, suggesting that stimulation of microglial expression of dynorphin A is a potential strategy in treatment of opioid addiction and abuse.

## Materials and Methods

### Drugs and Reagents

BAA was purchased from Zelang Bio-Pharmaceutical (Nanjing, China) with a purity no less than 98% determined by manufacturer with high performance liquid chromatography. Morphine hydrochloride, minocycline, and pentobarbital sodium were obtained from the Northeast Pharmaceuticals Group (Shenyang, China), Yuanye Biotech (Shanghai, China), and Sinopharm Chemical Reagent Co., (Shanghai, China), respectively. Both 5′-guanidinonaltrindole (GNTI) and naloxone hydrochloride were from Sigma-Aldrich (St. Louis, MO, United States). Furthermore, the rabbit polyclonal antiserum neutralizing dynorphin A was purchased from Phoenix Pharmaceuticals (Burlingame, CA, United States). The antiserum was specific to dynorphin A (100%), but not to dynorphin B (0%), β-endorphin (0%), α-neo-endorphin (0%) or leu-enkephalin (0%) according to the manufacturer′s datasheet. Its specificity was also validated by the antigen absorption test from other laboratories (Wakabayashi et al., 2010; Yamada et al., 2013). All the drugs and reagents were dissolved or diluted in 0.9% normal saline.

### Experimental Animals

Adult male Swiss mice (8–9 weeks and 20–25 g bodyweight) were purchased from the Shanghai Experimental Animal Institute for Biological Sciences (Shanghai, China). The animals were maintained in a 12-h light/dark cycle (light period 7:00 a.m.—7:00 p.m.) with free access to food and water at standard room temperature (22 ± 2°C) in the Shanghai Jiao Tong University Experimental Animal Center (Shanghai, China). All mice were acclimatized 3–5 days before the experiments. Mice (n = 10–12 per group) were randomly assigned and the behavior tests were performed in a blind manner. All housing conditions and experimental procedures were approved by the Animal Care and Welfare Committee of Shanghai Jiao Tong University (Shanghai, China).

### Induction of Morphine-Induced Withdrawal Symptoms in Mice

The paradigm in induction of morphine-induced withdrawal symptoms was performed as established previously (Goeldner et al., 2011; Bobzean et al., 2019). Briefly, morphine was administered in mice with escalating doses (5, 10, 20, 40, 80, and 100 mg/kg) by twice-daily subcutaneous injections at 10:00 a.m. and 4:00 p.m. for six consecutive days. On the seventh day, mice received a single subcutaneous injection of morphine (100 mg/kg) at 10:00 a.m., followed by an intraperitoneal injection of naloxone (5 mg/kg) 4 h later. Naloxone, by blocking opioid receptors, can expedite morphine withdrawal symptoms and is widely applied in the morphine addiction studies. The withdrawal symptoms, including shakes, jumps, genital licks, fecal excretion and loss of body weight, were observed and recorded for 30 min immediately after naloxone injection. To test the effect of BAA on morphine-induced withdrawal symptoms, mice received a single bolus BAA injection (30, 100, or 300 μg/kg) 40 min prior to the intraperitoneal injection of naloxone.

### Conditioned Place Preference Apparatus and Paradigm

CPP is a widely used model to assess the reinforcing effect of drug abuse in laboratories (Wu et al., 2016). As like other addictive drugs, morphine-induced CPP expression is considered to constitute a part of the addiction process associated with the opioid reinforcing properties. The apparatus in the CPP test consists of three compartments: two equal-sized chambers (25 × 25 × 40 cm) with a connecting white protruded chamber (null compartment, 25 × 5 × 40 cm) separated by a removable door. To distinguish each other, one of the main chambers was decorated with black walls and a striped floor, while the other one was with black and white striped wall and round dot floor. The environmental lighting was adjusted to exclude baseline preference. The apparatus was kept in a quiet room and dim 40 lx illumination (Marszalek-Grabska et al., 2018).

The 10-day scheduled CPP paradigm included three distinct phases: preconditioning, conditioning, and post-conditioning (Khaleghzadeh-Ahangar and Haghparast, 2015; Khaleghzadeh-Ahangar and Haghparast, 2017). The preconditioning phase started with a 3-day twice-daily (10:00 a.m. and 4:00 p.m.) mouse handling with the cupping open gloved hand method (Gouveia and Hurst, 2017). On Day 4, each mouse was placed into the null compartment with full access to the entire apparatus for 15 min. The time spent in each chamber was recorded by a 3CCD camera (Panasonic Inc., Japan) and analyzed using the EthoVision XT 8.0 (Noldus Information Technology Co., China) to determine the baseline preference. Animals that spent more than 450 s in any of the three chambers were excluded from the experiment. During the conditioning phase, mice underwent 5 days of morphine (10 mg/kg) or saline (10 ml/kg) alternatively subcutaneous injections, with a 6-h interval (between 10:00 a.m. and 4:00 p.m.) and included ten 45-min sessions in a five-day schedule. On day 5, 7, and 9 of the conditioning phase, mice were treated with morphine in the morning and immediately confined to the morphine-paired chamber for 45 min and received saline in the afternoon and then put into the saline-paired chamber for 45 min. On day 6 and 8, the injection sequence of morphine and saline was changed. Morphine-induced CPP in mice was tested by being allowed with free access to all three compartments for 15 min in the post-conditioning phase (on Day 10). The conditioning score was expressed by the time spent in the drug-paired chamber minus that in the saline-paired chamber. To determine the influence of BAA on morphine-induced CPP acquisition and expression, BAA was administration 30 min prior to morphine injection during the conditioning phase and 50 min prior to the post-conditioning phase, respectively.

### Morphine-Induced Locomotor Sensitization

The locomotor sensitization is a phenomenon that repeated administration of opioids can induce a progressive and long-lasting enhancement in behavioral response, which is associated with relapse and compulsive drug-seeking (Zhang et al., 2003). The methods for the behavioral sensitization in mice were described previously (Cordonnier et al., 2007). Mice were placed into a locomotor detection chamber (40 × 40 × 35 cm) under a video tracking system, and the data were analyzed automatically using ANY-maze. The procedure of the development of morphine-induced behavioral sensitization included habituation phase (Day 1–3) and morphine-induced behavioral sensitization phase (Day 4–8). In the habituation phase (Day 1–3), all mice were injected normal saline (10 ml/kg) and placed into the test apparatus for 3 days (1 h per session). In the morphine-induced behavioral sensitization phase (Day 4–8), mice were injected with saline (10 ml/kg) or morphine (10 mg/kg), and then placed into the test apparatus, where their locomotion was recorded for 1 h/day for 5 days. Mice received subcutaneous BAA injection (300 μg/kg) 20 min before morphine injection for 5 days (Day 4–8).

### Intracerebroventricular Catheterization and Injection in Mice

**FIGURE 1:**
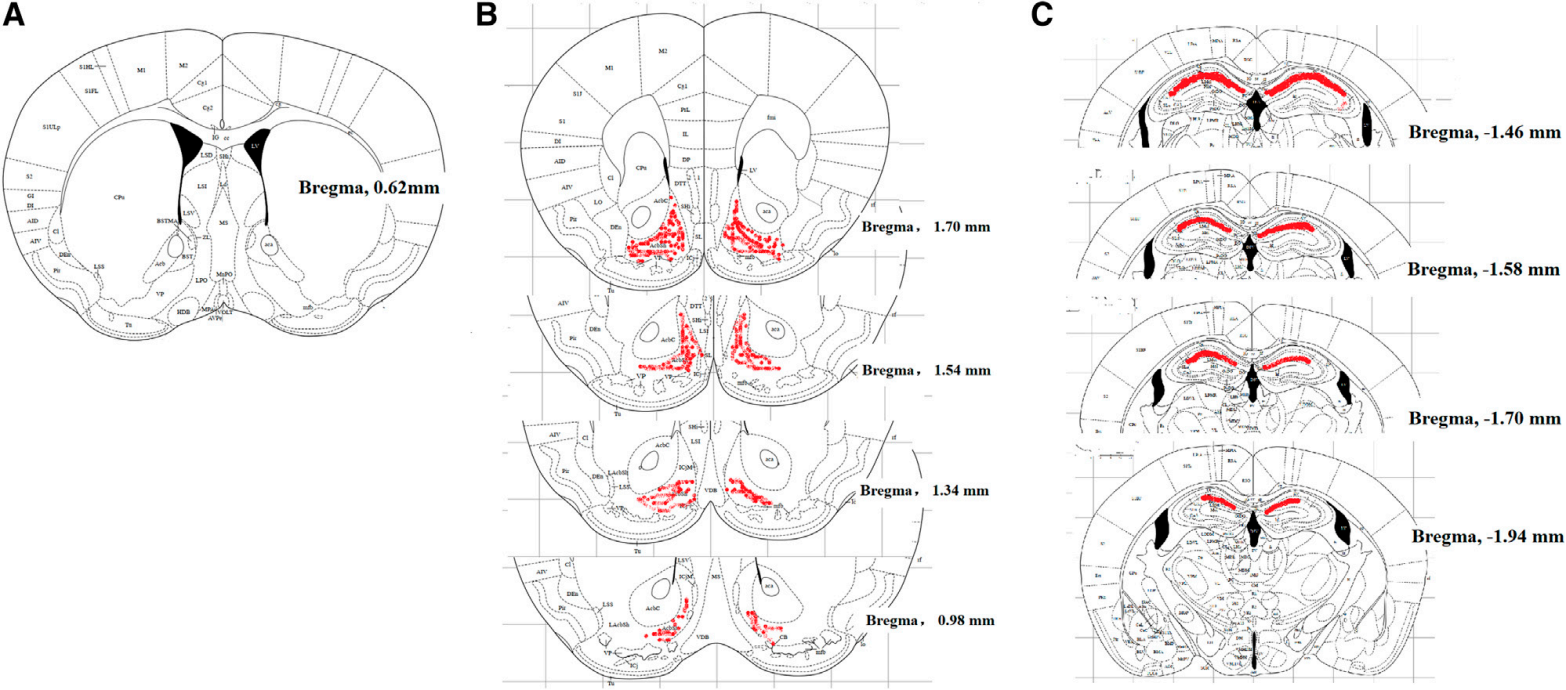
Localization of mouse cerebroventricle [in blank, **(A)**], nucleus accumbens shell [NAcSh, in red, **(B)**] and hippocampal CA1 [in red, **(C)**], displayed in the coronal plane. The cerebroventricle is approximately 1.0 mm lateral to the sagittal suture and 0.6 mm caudal from the bregma; the NAcSh is 1.70 to 0.98 mm from the bregma; hippocampal CA1 is −1.46 to −1.94 mm from the bregma.

For intracerebroventricular catheterization, mice were anesthetized by intraperitoneal injection of 1.5% pentobarbital sodium and positioned in a stereotaxic instrument (Stoelting Company, Wood Dale, IL, United States). The surgical site was shaved and sterilized with 70% ethanol and a 1.5 cm incision was made to expose the skull. A 22-gauge stainless steel cannula was directed to 1.0 mm lateral and 0.6 mm caudal to bregma and inserted 3 mm deep according to the mouse brain stereotaxic coordinates (Figure 1A). Dental cement was applied to adhere the cannula to the skull. The incision was sutured and the cap of cannula was covered. Animals were returned to their cages and allowed recovery at least for three days. The drug was administrated slowly over 3 min in a 6-μL volume through the planted cannula, using an insulin needle mated with a 10-μL microsyringe via a polyethylene tube (Kim et al., 1998; Lenard and Roerig, 2005; Glascock et al., 2011; Kim et al., 2016). It is noted that the ventricular injection may be a limit as its injection volume more than 2 μL could affect the behaviors of the animals. To verify the causal relationship between the microglial expression of dynorphin A in the brain and BAA-inhibited withdrawal signs in morphine-treated mice, the microglial metabolic inhibitor minocycline (10 μg) (Neigh et al., 2009), dynorphin A antiserum (1:30 dilution) (Li et al., 2016a) and KOR antagonist GNTI (5 μg) (Loh et al., 2017) were intracerebroventricularly injected into mice separately.

### RNA Isolation and Real-Time Quantitative Polymerase Chain Reaction

The total RNA was isolated from mouse nucleus accumbens (NAc) and hippocampus using the TRIzol reagent (Invitrogen, Carlsbad, CA, United States) and reversely transcribed into cDNA using the ReverTraAce RT-qPCR kit (Toyobo Co., Osaka, Japan) according to the manufacturers’ instructions. qPCR was performed with a Mastercycler ep realplex (Eppendorf, Hamburg, Germany) using the Realmaster Mix (SYBR Green I, Toyobo, Japan). The forward and reverse primer sequences were 5′-ATG ATG AGA CGC CAT CCT TC-3′ and 5′-TTA ATG AGG GCT GTG GGA AC-3′ for prodynorphin, which was designed by Premier 6 (version 6.0, Premier Biosoft, San Francisco, United States); and 5′-CCA AGG TCA TCC ATG ACG AC-3′ and 5′-TCC ACA GTC TTC TGA GTG GC-3′ for GAPDH (Reiss et al., 2017). The fold change was calculated using the 2^−△△Ct^ method after normalization to level of GAPDH mRNA (Huang et al., 2017b).

### Measurement of Dynorphin A

Mouse NAc and hippocampus were obtained and immediately frozen in liquid nitrogen and stored an –80°C until use. Tissues were homogenized at 4,000 rpm for 15 s with a homogenizer (Fluko Equipment Co., Shanghai, China) in 10 mM Tris-HCl (pH 7.4) and centrifuged at 1,500 rpm at 4°C for 15 min. The total protein concentrations in NAc and hippocampus were determined by a standard bicinchoninic acid protein assay (Beyotime Institute of Biotechnology, Jiangsu, China) and dynorphin A was assayed using a commercialized fluorescence enzyme-linked immunosorbent assay (ELISA) kit (Phoenix Pharmaceuticals, Burlingame, CA, United States) according to the operation manual (Leitermann et al., 2004; Nocjar et al., 2012).

### Immunofluorescence Staining

Double immunofluorescence labeling of dynorphin A and cellular biomarkers of microglia, astrocytes, and neurons in mouse NAc and hippocampus was carried out using a TCS SP8 confocal microscope (Leica Microsystems, Wetzlar, Germany) according to the previously published method with minor modifications. Mice were deeply anesthetized by intraperitoneal 1.5% pentobarbital sodium (5 ml/kg), and intracardially perfusion with 20 ml of 0.9% saline, followed by 20 ml of 4% paraformaldehyde. The brain was dissected and fixed in the 4% paraformaldehyde for 12 h at 4°C. Paraformaldehyde was then removed with phosphate buffered saline (PBS) and the brain was dehydrated with the gradient sucrose solutions (10%, 20% and 30% diluted with PBS) at 4°C. The dehydrated brain was embedded in the optimal cutting temperature embedding agent (Leica Microsystems) and cut into 30-μm-thick transverse sections with a sliding microtome. The frozen sections were incubated in 10% goat serum (v/v) and 0.5% Triton X-100 (v/v) for 1 h at the room temperature and then incubated at 4°C for 24 h with different primary antibodies. The primary antibodies included an anti-dynorphin A antibody (1:100; rabbit polyclonal; Phoenix Pharmaceuticals) and cellular markers, i.e., anti-Iba-1 (1:100; mouse monoclonal; Millipore, Darmstadt, Germany) for microglia, anti-GFAP (1:100; mouse monoclonal) for astrocytes, and anti-NeuN (1:60; mouse polyclonal; Millipore) for neurons. After washing with PBS, the sections were incubated for 1 h at 37°C with the Alexa-555-conjugated goat anti-rabbit secondary antibody for dynorphin A and the Alexa-488-conjugated goat anti-mouse secondary antibody for microglia, astrocytes or neurons (Qi et al., 2018). Expression of dynorphin A, Iba-1, GFAP, and NeuN was visualized in the shell of nucleus accumbens (NAcSh) (from bregma 1.70 mm to 0.98 mm, according to the mouse brain stereotaxic coordinates, Figure 1B) and hippocampal CA1 (from bregma −1.46 to −1.94 mm, according to the mouse brain stereotaxic coordinates, Figure 1C) under a confocal microscope. To quantify the relative intensity of dynorphin A in Iba-1-, GFAP- or NeuN-immunopositive cells in NAcSh and hippocampal CA1, the images were acquired at a 10× or 30× magnification. The background fluorescence was normalized and only immunofluorescent intensity from positively stained areas were included using the low and high thresholds. A co-localization analysis was performed using the ImageJ software with a co-localization finder to generate images in which the co-localized pixels appeared as white. All surface areas in each group were measured following the same setup configurations at the same time. The averaged value of the immunolabeled surface area was recorded as the positive immunofluorescence area from three nonadjacent sections of NAcSh or hippocampal CA1. Data were calculated from six mice of each group.

### Statistical analysis

For the dose-response curve analysis, the parameters, i.e., the minimum effect, half-effective dose (ED_50_), E_max_ and Hill coefficient (n), were calculated by fitting nonlinear least-squares curves to the relation Y = a + bx, where x = [D]^n^/(ED_50_^n^ + [D]^n^). The values of ED_50_ and b (E_max_) were projected by yielding a minimum residual sum of squares of deviations from the theoretical curve (Zhang et al., 2013).

The data were summarized as means ± standard error of the mean (S.E.M.). The statistical significance was evaluated by unpaired and two-tailed Student *t*-test, one-way or repeated-measures two-way analysis of variance (ANOVA) using the Prism (version 7.00, GraphPad Software Inc., San Diego, CA, United States). The ANOVA analysis was performed based on the assumptions of normal distribution and variance consistency verified by residual plots. The post-hoc Student-Newman-Keuls test was used when the effect of the drug (for the one-way ANOVA, the factor was drug; for the two-way ANOVA, the factors were drug, time and their interaction) was observed to be statistically significant. The probability values were two-tailed and the statistical significance criterion value was 0.05.

## Results

### Bulleyaconitine A Attenuates Naloxone-Induced Withdrawal Symptoms in Morphine-Treated Mice

**FIGURE 2:**
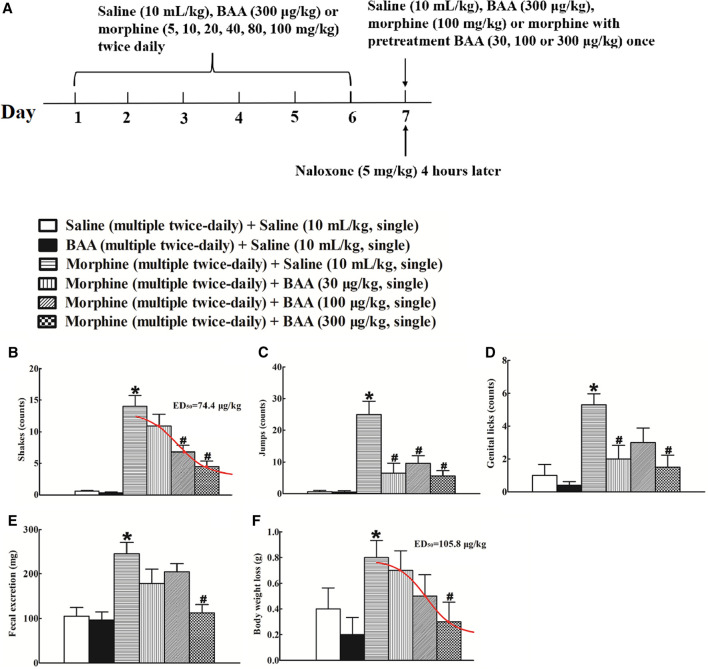
Inhibitory effects of subcutaneous (sc) injection of bulleyaconitine A on naloxone-induced withdrawal symptoms in morphine-treated mice **(A),** the timeline of the experiment procedure), including shakes **(B)**, jumps **(C)**, genital licks **(D)**, fecal excretion **(E)** and body weight loss **(F)**. Mice were subjected to bi-daily subcutaneous injections of normal saline (10 ml/kg), BAA (300 μg/kg) or morphine (an escalating doses of 5, 10, 20, 40, 80 and 100 mg/kg) for 6 days. On Day 7, mice received an intraperitoneal injection of naloxone (5 mg/kg) 4 h post last injection of morphine (100 mg/kg) to induce withdrawal signs, which were observed immediately for 30 min. For the BAA inhibitory effects, mice received a single injection of saline (10 ml/kg) or BAA (30, 100 or 300 μg/kg) 40 min prior to naloxone intraperitoneal injection. The dose-response data were best projected by the nonlinear least squares methods in **B** and **F**. The data are presented as means ± S.E.M (n = 10 per group). *^, #^ *p* < 0.05 compared with the saline control mice and morphine-treated mice, by one-way ANOVA followed by the post-hoc Student-Newman-Keuls test.

Six groups of mice (n = 10 per group) were subjected to bi-daily subcutaneous injections of normal saline (10 ml/kg), BAA (300 μg/kg) or morphine (escalating doses of 5, 10, 20, 40, 80, and 100 mg/kg) for 7 days. On Day 7, mice received an intraperitoneal injection of naloxone (5 mg/kg) 4 h post last injection of saline, BAA, or morphine (100 mg/kg), and their withdrawal symptoms were observed immediately for 30 min. For the BAA inhibitory effects, mice received a single injection of saline (10 ml/kg) or BAA (30, 100, or 300 μg/kg) 40 min prior to the intraperitoneal naloxone injection. The experiment procedure is shown in Figure 2A. Intraperitoneal naloxone injection did not induce any abnormal behaviors in bi-daily saline- or BAA-treated mice. In contrast, naloxone in bi-daily morphine injected mice induced significant withdrawal symptoms, including shakes [F (5, 54) = 23.13, *p* < 0.05; Figure 2B], jumps [F (5, 54) = 13.50, *p* < 0.05; Figure 2C], genital licks [F (5, 54) = 6.578, *p* < 0.05; Figure 2D], fecal excretion [F (5, 54) = 7.284, *p* < 0.05; Figure 2E], and body weight loss [F (5, 54) = 2.356, *p* < 0.05; Figure 2F]. In addition, pretreatment with a single subcutaneous BAA injection (30, 100 and 300 μg/kg) dose-dependently attenuated naloxone-induced withdrawal signs in bi-daily morphine-treated mice, with a maximal inhibition of around 70–100% in each sign. The dose-response analyses were performed after data transformation, yielding ED_50_ values of 74.4 μg/kg in shakes (Figure 2B), and 105.8 μg/kg in body weight loss, respectively (Figure 2F).

### Bulleyaconitine A Attenuates Morphine-Induced Conditioned Place Preference Acquisition and Expression

**FIGURE 3:**
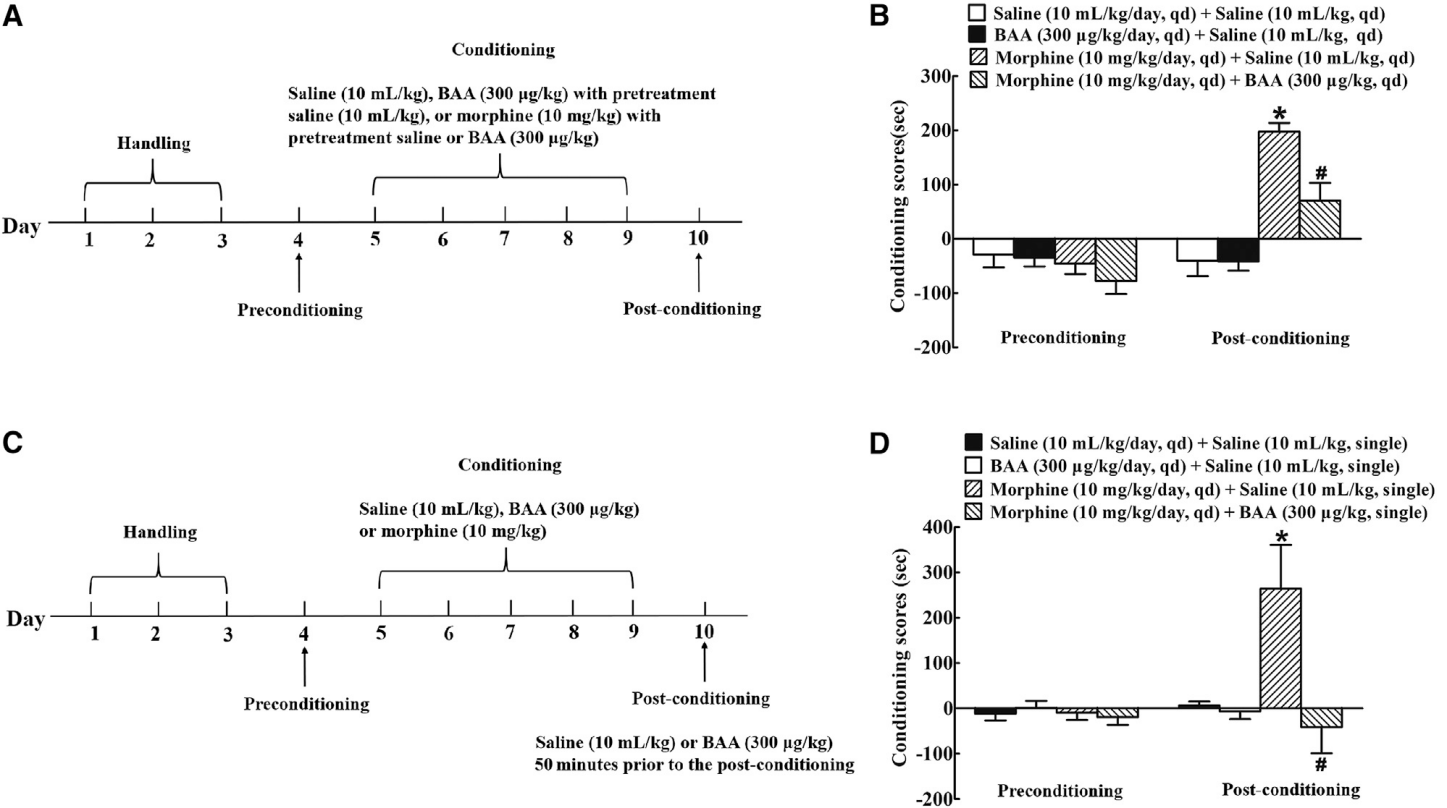
Inhibitory effects of subcutaneous (sc) injection of bulleyaconitine A (BAA, 300 μg/kg) on morphine-induced conditioned place preference (CPP) acquisition **(A,B)** and expression **(C,D)** in mice. For morphine-induced CPP acquisition, mice were treated with saline (10 ml/kg) or BAA (300 μg/kg) 30 min before each normal saline (10 ml/kg/day), BAA (300 μg/kg/day) or morphine (10 mg/kg/day) treatment during the conditioning phase. For morphine-induced CPP expression, mice were subjected to alternatively daily subcutaneous injections of normal saline (10 ml/kg/day), BAA (300 μg/kg/day), or morphine (10 mg/kg/day) for 5 days followed by a single subcutaneous injection of saline (10 ml/kg) or BAA (300 μg/kg) 50 min before the post-conditioning phase. Results are presented as means ± S.E.M. (n = 11 or 12 per group). *p < 0.05, compared to the saline control and morphine CPP control groups, respectively, by one-way ANOVA followed by the post-hoc Student-Newman-Keuls test.

To assess the inhibitory effect of BAA on morphine-induced CPP acquisition, four groups of mice (n = 12 per group) were subjected to the preconditioning phase of three days and conditioning phase of five days. The alternative daily subcutaneous injections of saline (10 ml/kg) or BAA (300 μg/kg) 30 min were prior to each morphine (10 mg/kg) or saline injection (10 ml/kg) during the conditioning phase (Figure 3A). There was no significant difference between the time spent in morphine-paired and saline-paired compartments during the preconditioning phase. Repeated morphine subcutaneous injections during the conditioning phase produced significant CPP acquisition, whereas saline or BAA did not show any CPP responses. Co-administrations of BAA (300 μg/kg) completely inhibited morphine-induced CPP acquisition [F (3, 40) = 21.42, *p* < 0.05, by one-way ANOVA followed by the post-hoc Student-Newman-Keuls test; Figure 3B].

To further determine the influence of BAA on morphine-induced CPP expression, four groups of mice (n = 12 per group) were subjected to alternative daily subcutaneous injections of normal saline (10 ml/kg), BAA (300 μg/kg) or morphine (10 mg/kg) for 5 days after the preconditioning phase of three days. On the 10th day, mice received a single subcutaneous injection of saline (10 ml/kg) or BAA (300 μg/kg) 50 min prior to the post-conditioning and the place preference test was conducted immediately afterward (Figure 3C). Bi-daily subcutaneous injections of morphine but not saline or BAA showed remarkable CPP expression, while pretreatment with a single subcutaneous BAA injection completely attenuated morphine-induced CPP expression [F (3, 44) = 6.043, *p* < 0.05, by one-way ANOVA followed by the post-hoc Student-Newman-Keuls test; Figure 3D].

### Bulleyaconitine A Suppresses Morphine-Induced Locomotor Sensitization Development

**FIGURE 4:**
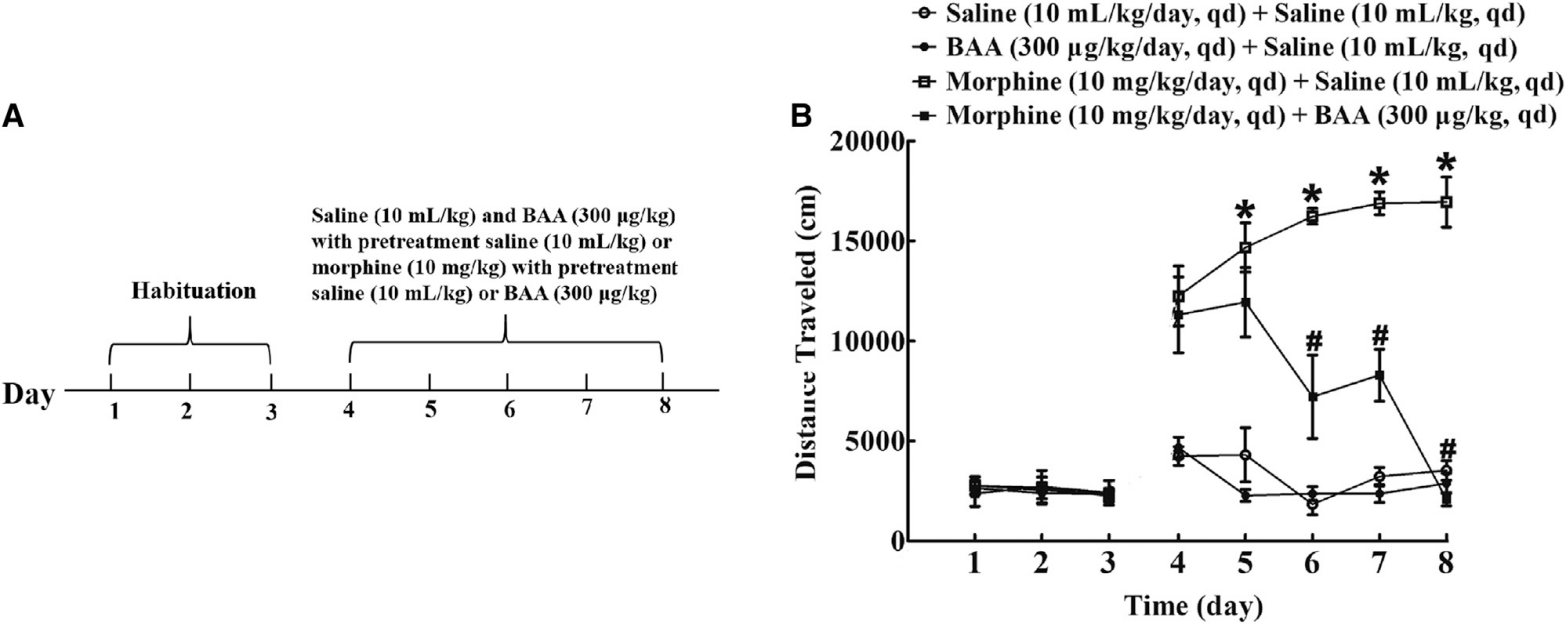
Inhibitory effects of subcutaneous (sc) injection of bulleyaconitine A (BAA) on morphine-induced locomotor sensitization **(A,B)**. Mice received subcutaneous injection of saline (10 ml/kg) or BAA (300 μg/kg) 20 min before normal saline (10 ml/kg), BAA (300 μg/kg) or morphine (10 mg/kg) injection for 5 days (Day 4–8) and the locomotor activity was recorded for 1 h each time point. Results are presented as means ± S.E.M. (n = 12 per group). *^, #^ *p* < 0.05 compared to the Day 4 and morphine group by repeated-measures two-way ANOVA followed by the post-hoc Student-Newman-Keuls test.

To investigate the inhibitory effect of BAA on morphine-induced locomotor sensitization development, four groups of mice (n = 12 per group) were subjected to the habituation phase (Day 1–3). After that, mice received a subcutaneous injection of saline (10 ml/kg) or BAA (300 μg/kg) 20 min before normal saline (10 ml/kg), BAA (300 μg/kg) or morphine (10 mg/kg) injection for 5 days (Day 4–8). The locomotion activity was then recorded for 1 h in each time point (Figure 4A). The 5-day BAA treatment did not significantly influence the locomotion activity compared to the saline control group. However, morphine treatment significantly increased the locomotion activity and the multi-daily treatment further significantly increased the travel distance [F (3, 155)_Day_ = 119.3, *p* < 0.05]. BAA co-treatment completely attenuated development of morphine-induced locomotor sensitization [F (4, 155)_Treatment_ = 3,398, *p* < 0.05, by repeated-measures two-way ANOVA followed by the post-hoc Student-Newman-Keuls test; Figure 4B].

### Bulleyaconitine A Specifically Stimulates Microglial Dynorphin A Expression in NAc and Hippocampus in Morphine-Multi-Daily Treated Mice

**FIGURE 5:**
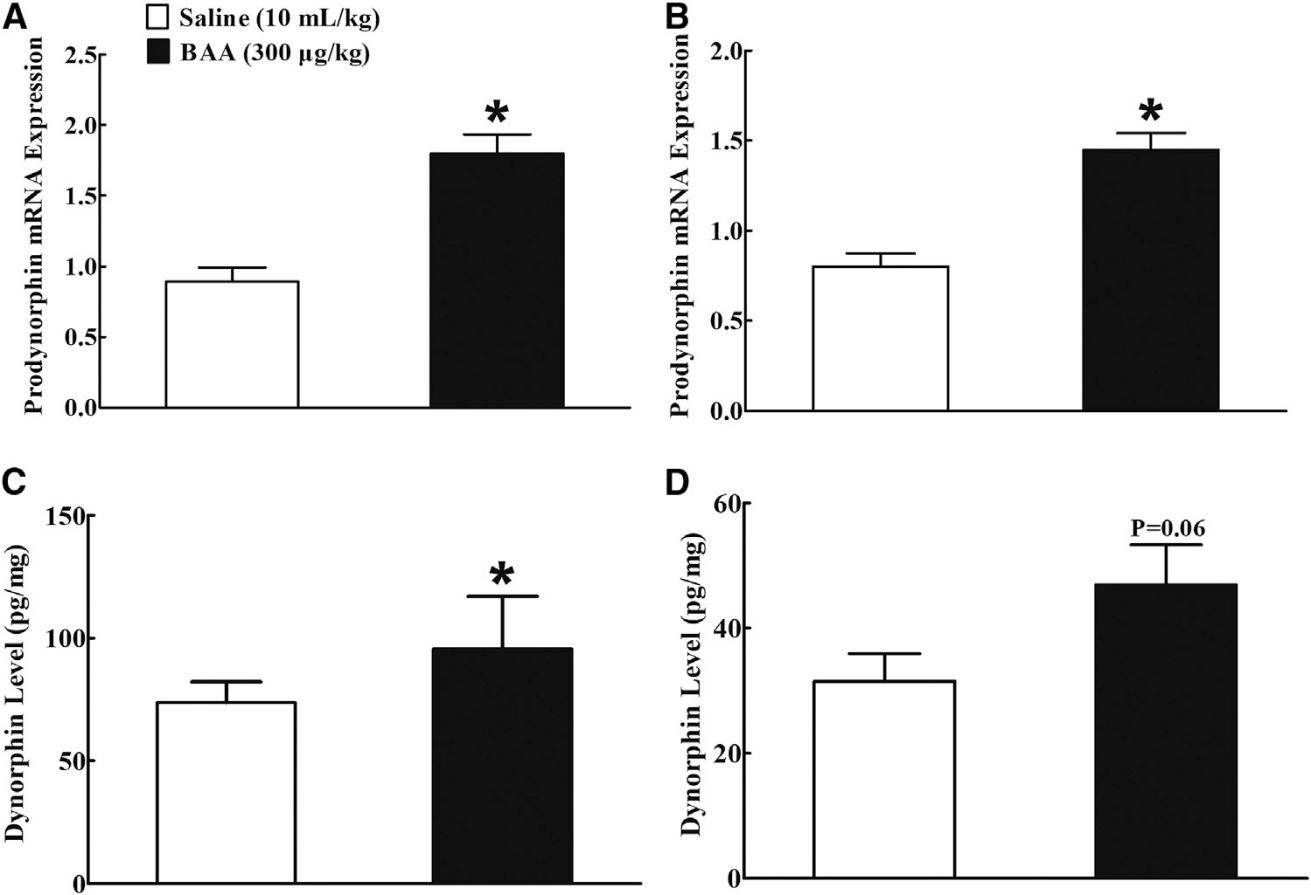
Stimulatory effects of subcutaneous (sc) injection of bulleyaconitine A (BAA, 300 μg/kg) on dynorphin A mRNA **(A,B)** and protein **(C,D)** levels in nucleus accumbens (NAc) and hippocampus in multiple daily morphine (10 mg/kg)-treated mice. Levels of dynorphin A mRNA and protein were measured by using qRT-PCR and fluorescent ELISA kit, respectively. The data are presented as mean ± S.E.M (n = 8–12 per group). *p < 0.05, compared to the saline control group, by unpaired and two-tailed Student t-test.

Two groups of mice (n = 10 per group) treated daily with morphine (10 mg/kg) for 5 days received a subcutaneous injection of normal saline (10 ml/kg) or BAA (300 μg/kg). Mice were sacrificed 1 h after the subcutaneous injection and NAc and hippocampus were obtained for the prodynorphin mRNA detection using qRT-PCR analysis. As shown, BAA treatment compared to the saline control group significantly increased prodynorphin gene expression by 1.9-fold in NAc Figure 5A) and 1.7- fold in hippocampus (*p* < 0.05, by unpaired and two-tailed Student t-test; Figure 5B). The stimulatory effects of BAA on expression of dynorphin A protein were also measured in NAc and hippocampus in the same mice using the commercial fluorescent ELISA kit. As exhibited, subcutaneous BAA significantly increased dynorphin A expression in NAc (Figure 5C) and hippocampus, compared to the saline control group (*p* < 0.05, by unpaired and two-tailed Student t-test; Figure 5D).

**FIGURE 6:**
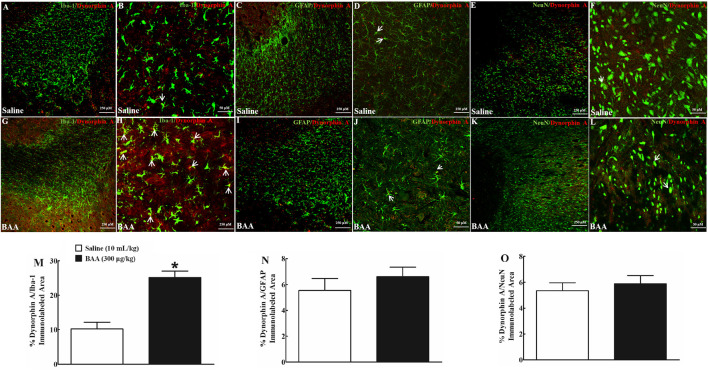
Specific stimulatory effects of subcutaneous (sc) injection of bulleyaconitine A (BAA, 300 μg/kg) on dynorphin A expression in microglia, but not in astrocytes or neurons in the shell of nucleus accumbens (NAcSh) in multiple daily morphine (10 mg/kg)-treated mice. Frozen sections of the NAcSh were obtained 1 h after a subcutaneous injection of saline (10 ml/kg) or BAA (300 μg/kg) and subjected to double immunofluorescence staining with dynorphin A/microglial marker Iba-1 **(A,B,G,H)**, dynorphin A/astrocytic marker GFAP **(C,D,I,J)** and dynorphin A/neuronal marker NeuN **(E,F,K,L)** under 10× magnification (The scale bar, 250 μm) and 30× magnification (The scale bar, 50 μm), respectively. The arrowheads indicate co-localization of dynorphin A with microglia, astrocytes or neurons. The co-localized areas of dynorphin A/Iba-1 (**M**), dynorphin A/GFAP (**N**) and dynorphin A/NeuN (**O**) were quantified at 10× magnification using the ImageJ software. The data are presented as mean ± S.E.M. (n = 6 per group). *p < 0.05, compared to the saline control group, by unpaired and two-tailed Student t-test.

**FIGURE 7:**
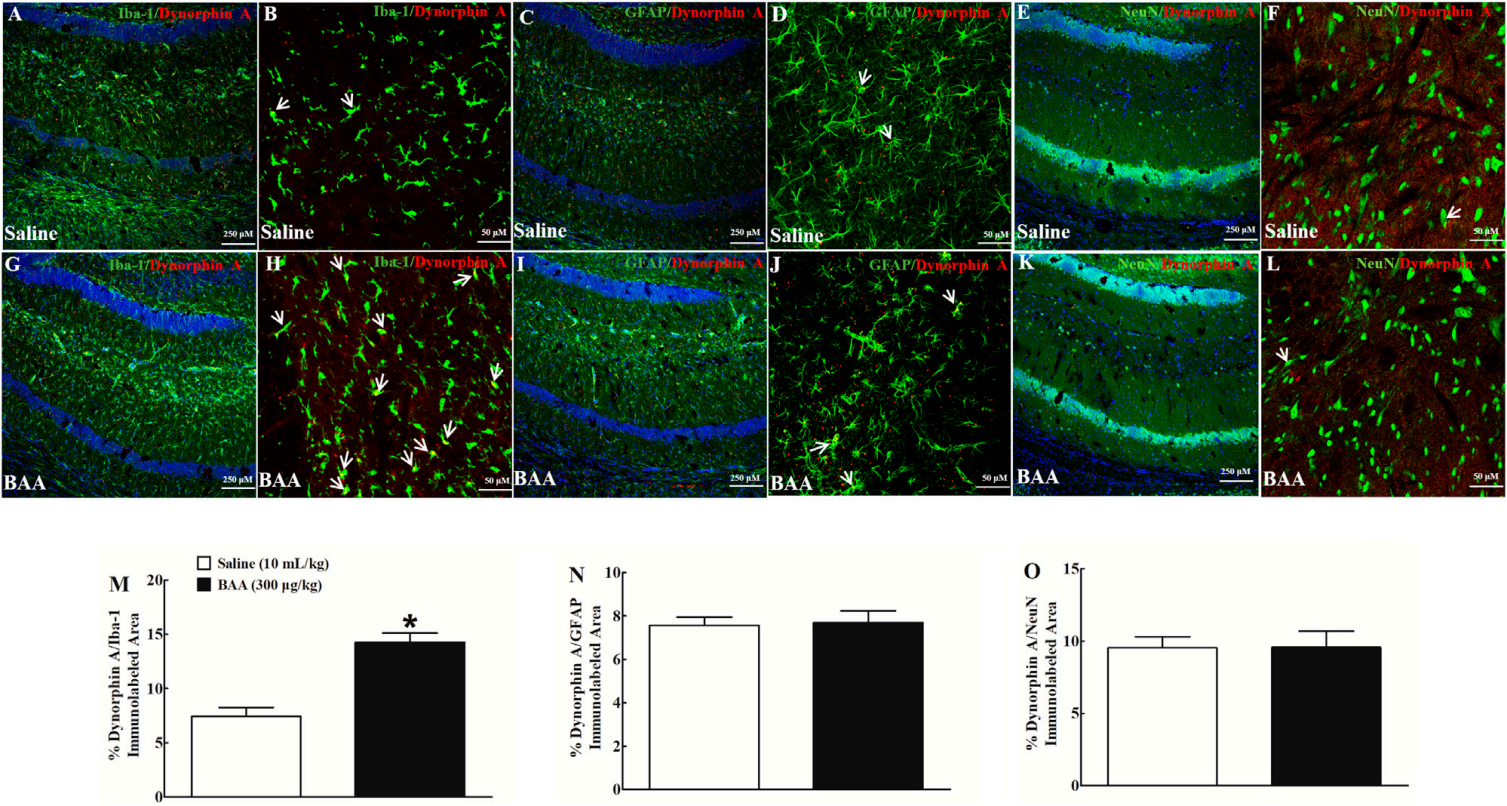
Specific stimulatory effects of subcutaneous (sc) injection of bulleyaconitine A (BAA, 300 μg/kg) on dynorphin A expression in microglia, but not in astrocytes or neurons in hippocampal CA1 in multiple twice-daily morphine (10 mg/kg)-treated mice. Frozen sections of hippocampal CA1 were obtained 1 h after a subcutaneous injection of saline (10 ml/kg) or BAA (300 μg/kg) and then subjected to double immunofluorescence staining with dynorphin A/microglial marker Iba-1 **(A,B,G,H)**, dynorphin A/astrocytic marker GFAP **(C,D,I,J)**, and dynorphin A/neuronal marker NeuN (**E,F,K,L**) under 10× magnification (The scale bar, 250 μm, DAPI was also co-labeled with the nucleus in blue) and 30× magnification (The scale bar, 50 μm), respectively. The arrowheads indicate co-localization of dynorphin A with microglia, astrocytes or neurons. The co-localized areas of dynorphin A/Iba-1 (**M**), dynorphin A/GFAP (**N**), and dynorphin A/NeuN (**O**) were quantified at 10× magnification using the ImageJ software. The data are presented as mean ± S.E.M. (n = 6 per group). *p < 0.05, compared to the saline control group, by unpaired and two-tailed Student t-test.

Dynorphin A is localized in neurons, astrocytes and microglia in the central nervous system (Wahlert et al., 2013; Ayrout et al., 2019). To verify cell types that specifically upregulate dynorphin A expression in NAcSh and hippocampal CA1 following BAA treatment, dynorphin A was immunofluorescence-labeled with the microglial cellular marker Iba-1, astrocytic cellular marker GFAP, and neuronal cellular marker NeuN. Two groups of mice (n = 6 per group) treated daily with morphine (10 mg/kg) for 5 days received a subcutaneous injection of saline (10 ml/kg) or BAA (300 μg/kg). Mice were then sacrificed 1 h after the subcutaneous injection and NAcSh and hippocampal CA1 were obtained for fluorescence immunostaining. As shown, dynorphin A was co-localized with Iba-1, GFAP and NeuN in NAcSh of saline-treated mice (Figures 6A–F). Subcutaneous BAA specifically increased co-labeling of dynorphin A/Iba-1 (Figures 6G,H) but not dynorphin A/GFAP (Figures 6I,J) or dynorphin A/NeuN (Figures 6K,L) at 10× and 30× magnification. Furthermore, the ImageJ software was used to quantify immunofluorescence intensity of dynorphin A with Iba-1, GFAP or NeuN at 10× magnification. Treatment with subcutaneous BAA significantly increased dynorphin A/Iba-1 by 2.4-fold compared to the saline control group (*p* < 0.05, by unpaired and two-tailed Student t-test; Figure 6M), but not dynorphin A/GFAP (Figure 6N) or dynorphin A/NeuN (Figure 6O). In addition, the same specific stimulatory effects of BAA on microglial dynorphin A expression were observed in hippocampal CA1 from the same mice as above (Figures 7A–L), with an increase in immunofluorescence intensity of dynorphin A/Iba-1 by 1.9-fold (*p* < 0.05, by unpaired and two-tailed Student t-test; Figure 7M), but not dynorphin A/GFAP (Figure 7N) or dynorphin A/NeuN (by unpaired and two-tailed Student t-test; Figure 7O).

### Microglial dynorphin a Expression Mediates Bulleyaconitine A-Inhibited Morphine Dependence

**FIGURE 8:**
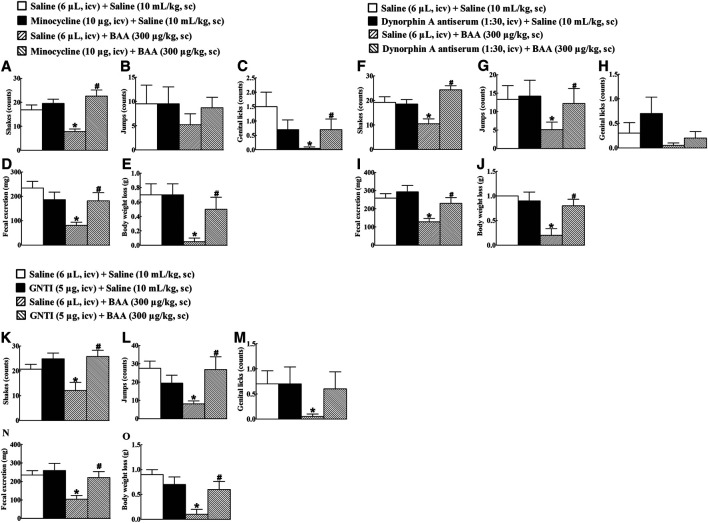
Blockade effects of intracerebroventricular (icv) injection of the microglial metabolic inhibitor minocycline **(A–E)**, specific dynorphin A antiserum **(F–J)**, and selective KOR antagonist GNTI (**K-O**) on subcutaneous (sc) injection of bulleyaconitine A (BAA)-attenuated withdrawal signs in morphine-treated mice. Naloxone-induced withdrawal symptoms included shakes **(A,F,K)**, jumps **(B,G,L)**, genital licks **(C,H,M)**, fecal excretion **(D,I,N)**, and body weight loss **(E,J,O)**. The data are presented as means ± S.E.M. (n = 10 per group). *^, #^ *p* < 0.05, compared to the saline control group and BAA group, respectively, by one-way ANOVA followed by the post-hoc Student-Newman-Keuls test.

To verify the causal relationship between the microglial expression of dynorphin A in the brain and the BAA-inhibited withdrawal signs in morphine-treated mice, the microglial metabolic inhibitor minocycline (Wu et al., 2002; Neigh et al., 2009; Kobayashi et al., 2013), dynorphin A antiserum (Li et al., 2016a), and KOR antagonist GNTI (Zhang et al., 2007; Liu et al., 2013) were intracerebroventricularly injected individually. Four groups of morphine-treated mice (n = 10 per group) received the first intracerebroventricular injection followed by the second subcutaneous injection of saline (6 μL) + saline (10 ml/kg), minocycline (10 μg) + saline (10 ml/kg), saline (6 μL) + BAA (300 μg/kg), or minocycline (10 μg) + BAA (300 μg/kg). The second subcutaneous injection was delivered 4 h post the first intracerebroventricular injection. Withdrawal signs were precipitated by the intraperitoneal injection of naloxone (5 mg/kg) 40 min after the subcutaneous injection. As shown in Figures 8A–E, subcutaneous BAA injection into morphine-treated mice significantly attenuated naloxone-induced withdrawal signs, including shakes, jumps, genital licks, fecal excretions, and body weight loss, whereas the intracerebroventricular minocycline failed to influence naloxone-induced morphine withdrawal responses in mice. However, pretreatment with intracerebroventricular minocycline nearly completely restored the systemic BAA-suppressed withdrawal symptoms.

Additional four groups of morphine-treated mice (n = 10 per group) received the first intracerebroventricular injection followed by the second subcutaneous injection of saline (6 μL) + saline (10 ml/kg), dynorphin A antiserum (1:30 dilution, 6 μL) + saline (10 ml/kg), saline (6 μL) + BAA (300 μg/kg), or dynorphin A antiserum (1:30 dilution, 6 μL) + BAA (300 μg/kg). The second subcutaneous injection was 30 min post the first intracerebroventricular injection. Withdrawal symptoms were precipitated by intraperitoneal injection of naloxone (5 mg/kg) 40 min after the subcutaneous injection. Subcutaneous BAA injection into morphine-treated mice attenuated naloxone-induced withdrawal signs. Intracerebroventricular injection of the dynorphin A antiserum did not significantly affect baseline morphine withdrawal symptoms, but reemerged naloxone-induced withdrawal symptoms from BAA inhibition (Figures 8F–J).

Further four groups of morphine-treated mice (n = 10 per group) received the same treatments as above except that the dynorphin A antiserum was replaced with GNTI (5 μg). As shown in Figures 8K–O, intracerebroventricular GNTI injection predominantly restored BAA-suppressed withdrawal symptoms in morphine-treated mice, although it did not significantly alter naloxone-induced withdrawal signs in saline-treated morphine-treated mice.

### Microglial Dynorphin A Expression Mediates Bulleyaconitine A-Inhibited Conditioned Place Preference Expression

**FIGURE 9:**
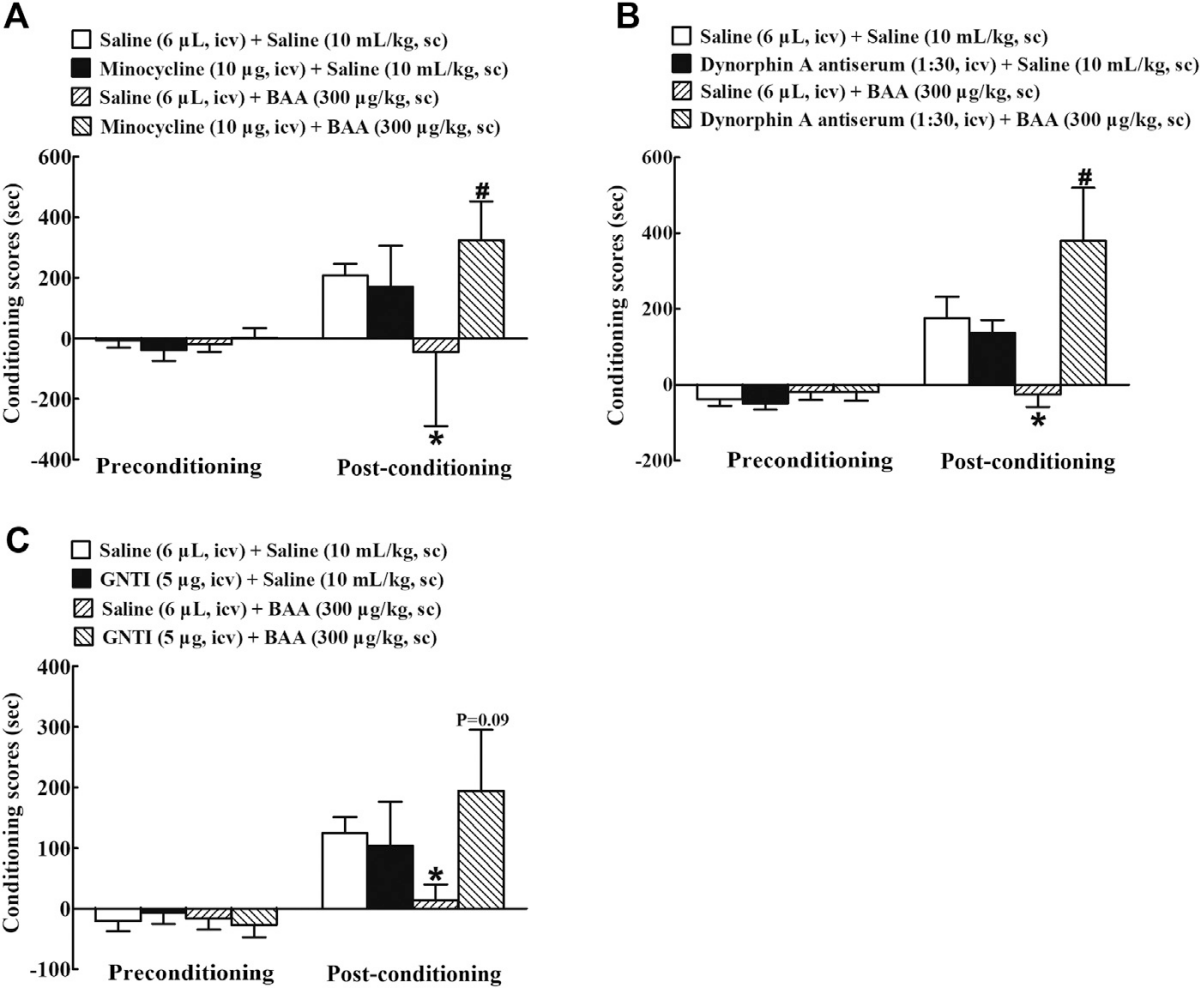
Blockade effects of intracerebroventricular (icv) injection of the microglial metabolic inhibitor minocycline **(A)**, specific dynorphin A antiserum **(B)**, and selective KOR antagonist GNTI **(C)** on a subcutaneous (sc) injection of bulleyaconitine A (BAA)-attenuated morphine conditioned place preference (CPP) expression in mice. The data are presented as means ± S.E.M. (n = 10 per group). *^, #^ *p* < 0.05, compared to the saline control group and BAA group, respectively, by one-way ANOVA followed by the post-hoc Student-Newman-Keuls test.

Minocycline, dynorphin A antiserum, and GNTI, given intracerebroventricularly, were applied to further determine whether microglial expression of dynorphin A in the brain contributed to BAA-inhibited morphine-induced CPP expression. Four groups of morphine-treated mice (n = 10 per group) were first intracerebroventricularly injected followed 4 h later by subcutaneously injected with saline (6 μL) + saline (10 ml/kg), minocycline (10 μg) + saline (10 ml/kg), saline (6 μL) + BAA (300 μg/kg), or minocycline (10 μg) + BAA (300 μg/kg). The place preference test was assessed 50 min subsequent to the subcutaneous injection. As shown in Figure 9A, subcutaneous BAA injection but not intracerebroventricular minocycline completely attenuated morphine-induced CPP expression. However, pretreatment with intracerebroventricular minocycline entirely restored BAA-suppressed CPP expression [F (3, 36) = 3.829, *p* < 0.05, by one-way ANOVA followed by the post-hoc Student-Newman-Keuls test].

Furthermore, four groups of morphine CPP mice (n = 10 per group) were first intracerebroventricularly injected 30 min later followed by subcutaneously injected with saline (6 μL) + saline (10 ml/kg), dynorphin A (1:30 dilution, 10 μL) + saline (10 ml/kg), saline (6 μL) + BAA (300 μg/kg), or dynorphin A (1:30 dilution, 10 μL) + BAA (300 μg/kg). The place preference test was assessed 50 min subsequent to the subcutaneous injection. Subcutaneous BAA injection but not intracerebroventricular dynorphin A antiserum totally inhibited expression of morphine-induced CPP. However, pretreatment with intracerebroventricular injection of dynorphin A antiserum completely attenuated BAA-suppressed morphine-induced CPP expression [F (3, 36) = 4.459, *p* < 0.05, by one-way ANOVA followed by the post-hoc Student-Newman-Keuls test; Figure 9B].

In addition, other four groups of morphine CPP mice (n = 10 per group) received the same treatment regimen as above except that the dynorphin A antiserum was replaced with GNTI (5 μg). As shown in Figure 9C, intracerebroventricular GNTI injection did not have any significantly inhibitory effects on morphine-induced CPP expression, but almost or totally restored the systemic BAA-suppressed withdrawal signs in morphine-treated mice (F (3, 36) = 1.330, *p* = 0.09, by one-way ANOVA followed by the post-hoc Student-Newman-Keuls test; Figure 9C).

## Discussion

Long-term use of morphine and other opioids induces addiction, including both physical and psychological dependences. In the present study, withdrawal symptoms were developed after bi-daily subcutaneous morphine injections into mice for 7 consecutive days, which was reflected by withdrawal signs (i.e., shakes, jumps, genital licks, fecal excretion, and body weight loss) following application of naloxone. In contrast, bi-daily subcutaneous BAA injections with a dose up to 300 μg/kg/day for 7 days did not induce any withdrawal symptoms, which is consistent with the previous finding in which daily subcutaneous BAA did not induce jumping responses following nalorphine challenge (Tang et al., 1986). Furthermore, a single subcutaneous BAA injection alleviated naloxone-induced withdrawal signs in morphine-treated mice. In a dose ranging between 30 and 300 μg/kg, BAA injection into morphine-treated mice caused a dose-related inhibition of abrupt withdrawal symptoms, typically shakes and body weight loss with ED_50_ values of 74.4 and 105.8 μg/kg respectively. Consistently, BAA analog lappaconitine was reported to alleviate morphine and cocaine physical dependence (Qu and Qu, 1994). On the other respect, a daily subcutaneous morphine injection but not BAA (300 μg/kg/day) for 5 consecutive days acquired remarkable CPP response with high conditioning scores. Co-administration of BAA significantly inhibited morphine-induced CPP acquisition during the conditioning phase. In addition, single subcutaneous BAA injection entirely abolished morphine-induced CPP expression in the post-conditioning phase. Furthermore, co-treatment with BAA entirely attenuated development of morphine-induced locomotor sensitization. All these results suggest that BAA does not induce physical or psychological dependence, but markedly alleviates morphine-induced withdrawal symptoms, CPP acquisition and expression, and locomotor sensitization. However, the hypothesis may be compromised because we just used male mice in this study and previous studies revealed that degrees of morphine-induced physical and psychological dependences varied with gender (Cicero et al., 2002; Mohammadian and Miladi-Gorji, 2019). Thus, future studies are needed to evaluate the anti-addictive effects of BAA in female animals.

Opiates, such as morphine and heroin, act at the mesolimbic dopamine pathway projecting from the ventral tegmental (VTA) to NAc (Patyal et al., 2012). Opiates drugs effectively stimulate dopamine release in the NAc within 1 h after intracerebroventricular injection or local administration into VTA (Murphy et al., 1996; Sebastian et al., 2016). Hippocampal input to NAcSh is important in rewarding behaviors (LeGates et al., 2018). Thus, it can be speculated that NAc and hippocampus, located around the cerebroventricular area, are important sites for development of drug addiction. Dynorphin A regulates the activity of dopamine neurons by acting on KORs in mesolimbic, NAcSh, prefrontal cortex and VTA that have been implicated in drug abuse liability (Meshul and McGinty, 2000; Volkow et al., 2009). In the current study, we explored whether the dynorphin A/KOR system in NAc and hippocampus was closely associated with BAA-attenuated naloxone-induced morphine withdrawal symptoms and CPP expression. Subcutaneous BAA injection into morphine-treated mice stimulated expression of dynorphin A in NAc and hippocampus at 1 h after injection, which was in agreement with the time-course of its anti-addictive effects. The results are parallel to the previous findings in which intrathecal and subcutaneous injection of BAA, bullatine A, and lappaconitine stimulated expression of dynorphin but not β-endorphin in the spinal cord (Li et al., 2016a; Li et al., 2016b).

The notion is further supported by the following interventional injections through the intracerebroventricular route, which is localized around NAc and hippocampus. It was previously reported that a single intravenous dynorphin A injection attenuated withdrawal symptoms of morphine dependence (Takemori et al., 1993). The present study demonstrated that intracerebroventricular dynorphin A antiserum injection totally eliminated systemic BAA-inhibited morphine withdrawal symptoms and CPP expression, although the injection volume more than 2 μL could affect the behaviors of the animals and might be a limitation. Moreover, the highly selective KOR antagonist GNTI, given intracerebroventricularly, also entirely eliminated the systemic BAA-inhibited morphine-induced withdrawal symptoms and CPP expression. These data are consistent with the previous studies showing that the KOR agonist salvinorin A punished self-administration of cocaine and remifentanil in monkeys (Freeman et al., 2014), and that addition of the KOR agonist U69,593 to fentanyl produced a proportion-dependent decrease in fentanyl self-administration in rats (Negus et al., 2008).

Drug abuse activates microglia to produce a large amount of inflammatory factors and affect synapse reconstruction, chemical changes in the neural signal transduction and phagocytosis of apoptotic neurons, and ultimately to regulate the dopamine reward-signaling pathway and enhance drug dependence and addiction (Kovacs, 2012; Garaschuk and Verkhratsky, 2019). However, recent studies also showed that microglia had an alternative activation or protective state to activate the anti-inflammatory cascades and exhibited neuroprotection and antinociception (Hu et al., 2012; Fan et al., 2015; Wu et al., 2017; Wu et al., 2018). Our present data provide additional evidence showing that BAA stimulated microglia to express dynorphin A for attenuation of the drug dependence and addiction. Subcutaneous BAA injection stimulated dynorphin A expression only in microglia and not in astrocytes and neurons in NAcSh and hippocampal CA1, similar to the previous findings in which injection of a BAA analog bullatine A specifically stimulated microglial (but not astrocytic or neural) expression of dynorphin A in the spinal cords of neuropathic rats (Huang et al., 2016). We further demonstrated that intracerebroventricular injection of the microglial metabolic inhibitor minocycline entirely blocked the systemic BAA-inhibited morphine-induced withdrawal symptoms and CPP expression. Consistently, the antinociceptive effects of BAA and its analogs bullatine A and lappaconitine were also blocked by the intrathecal injection of minocycline in the rat models of pain hypersensitivity (Huang et al., 2016; Sun et al., 2018; Huang et al., 2020b). These results highlight that stimulation of microglia expression and release of dynorphin A, in contrast to proinflammatory cytokines and neurotrophins, inhibited morphine-induced withdrawal symptoms and CPP expression as well as blocked pain hypersensitivity.

The main treatment option of the opioid addiction is detoxification and relieves withdrawal symptoms; for this purpose, methadone substitution is most commonly used. This treatment, however, is just provided to inpatients in well-equipped management institutions due to its own physical dependence and other adverse reactions (Kampman and Jarvis, 2015; Tran et al., 2017). On the other respect, psychotherapy of addiction should be emphasized conceptually. Only few patients are unfortunately willing to undergo drug addiction program, while many patients are still carving for addictive substances after improving withdrawal symptoms, which eventually leads to relapse (Arevalo et al., 2008). Thus, approaches at the current time to solve opioid addiction, especially psychological dependence, are limited and treatment strategy with novel mechanisms of actions is therefore, urgently needed. Our current study did provide a solid pharmacological base for BAA to alleviate morphine-induced withdrawal symptoms, CPP acquisition and expression, and locomotor sensitization in mice through the mechanism of microglial expression of dynorphin A. Moreover, oral administration of lappaconitine over 6 days reduced or eliminated withdrawal symptoms, like yawing, shedding tears, chilliness, mydriasis and restlessness in drug addict patients whose duration of heroin or opium addiction ranged from 1 to 4 years (Qu and Qu, 1994). Taken together, all these findings suggest that BAA is a promising clinical development candidate for treatment of opioid addiction, especially the psychological dependence. The data also indicate that targeting of microglial expression and secretion of dynorphin A is a potentially novel strategy in treatment of opioid drug addiction.

## Data Availability

The original contributions presented in the study are included in the article/Supplementary Material, further inquiries can be directed to the corresponding author.
